# Histological Changes in Severe Diabetic Fetopathy: An Autopsy Case Report

**DOI:** 10.7759/cureus.4199

**Published:** 2019-03-06

**Authors:** George S Stoyanov, Ina Kobakova, Lyuben Stoev, Hristo Popov, Savi R Shishkov, Kameliya Bratoeva

**Affiliations:** 1 General and Clinical Pathology, Forensic Medicine and Deontology, Medical University, Varna, BGR; 2 Endocrinology, Medical University – Varna "Prof. Dr. Paraskev Stoyanov", Varna, BGR; 3 Physiology and Pathophysiology, Medical University – Varna "Prof. Dr. Paraskev Stoyanov", Varna, BGR

**Keywords:** diabetic fetopathy, steatosis, langerhans amyloidosis, fetal hypertension, gestational diabetes

## Abstract

Maternal diabetes is one of the most common and dangerous risk factors during pregnancy, as often there are no generalized signs. Diabetic fetopathy is a severe, poorly defined complication of gestational diabetes or preexisting maternal diabetes mellitus, with an ill-defined histological spectrum of changes. Herein we report a case of severe diabetic fetopathy diagnosed upon autopsy of a recently miscarried fetus. On histology, the liver revealed severe generalized macrovesicular steatosis and number of small cysts. The pancreas revealed not only Langerhans isle hyperplasia, but also Langerhans amyloidosis, evident of the severity of maternal diabetes and fetal hyperglycemia. The adrenal glands revealed hyperplasia in zona glomerulosa, due to aldosterone overproduction, evident of fetal hypertension. The current case is an extreme example of an undiagnosed and untreated gestational diabetes mellitus. The severity of histological changes, in this case, is suggestive of further extension of the diagnostic criteria of diabetic fetopathy to include more subtle changes that can be observed clinically and even a combination of maternal-newborn factors.

## Introduction

Diabetic fetopathy is a severe fetal complication of gestational diabetes or preexisting maternal diabetes mellitus [1,2]. The condition is often poorly defined and the only key component often mentioned is fetal macrosomia, with clinical complications such as hypoglycemia, hypokalemia with cardiac cycle disturbances and others [1,3].

The range of histological changes is ill-defined as with the stabilization of the condition of the fetus postpartum, most of them tend to regress [2,4,5].

Herein we report a case of severe diabetic fetopathy diagnosed upon autopsy of a recently miscarried fetus.

## Case presentation

A 40th gestational week female miscarried fetus was presented for autopsy in the Department of General and Clinical Pathology, St. Marina University Hospital, Varna, Bulgaria. The mother, 23 years old and otherwise healthy, had one previous pregnancy which concluded with elective abortion. There was no rhesus factor incompatibility. Prior to the miscarriage, the mother had not felt any movement of the fetus for 24 hours. Clinically during the miscarriage, a wrapped and tightened umbilical cord was observed around the neck of the fetus. The autopsy was carried out 24 hours after the miscarriage, a total of 48 hours after the fetal death, no placental specimen was sent for investigation.

**Figure 1:**
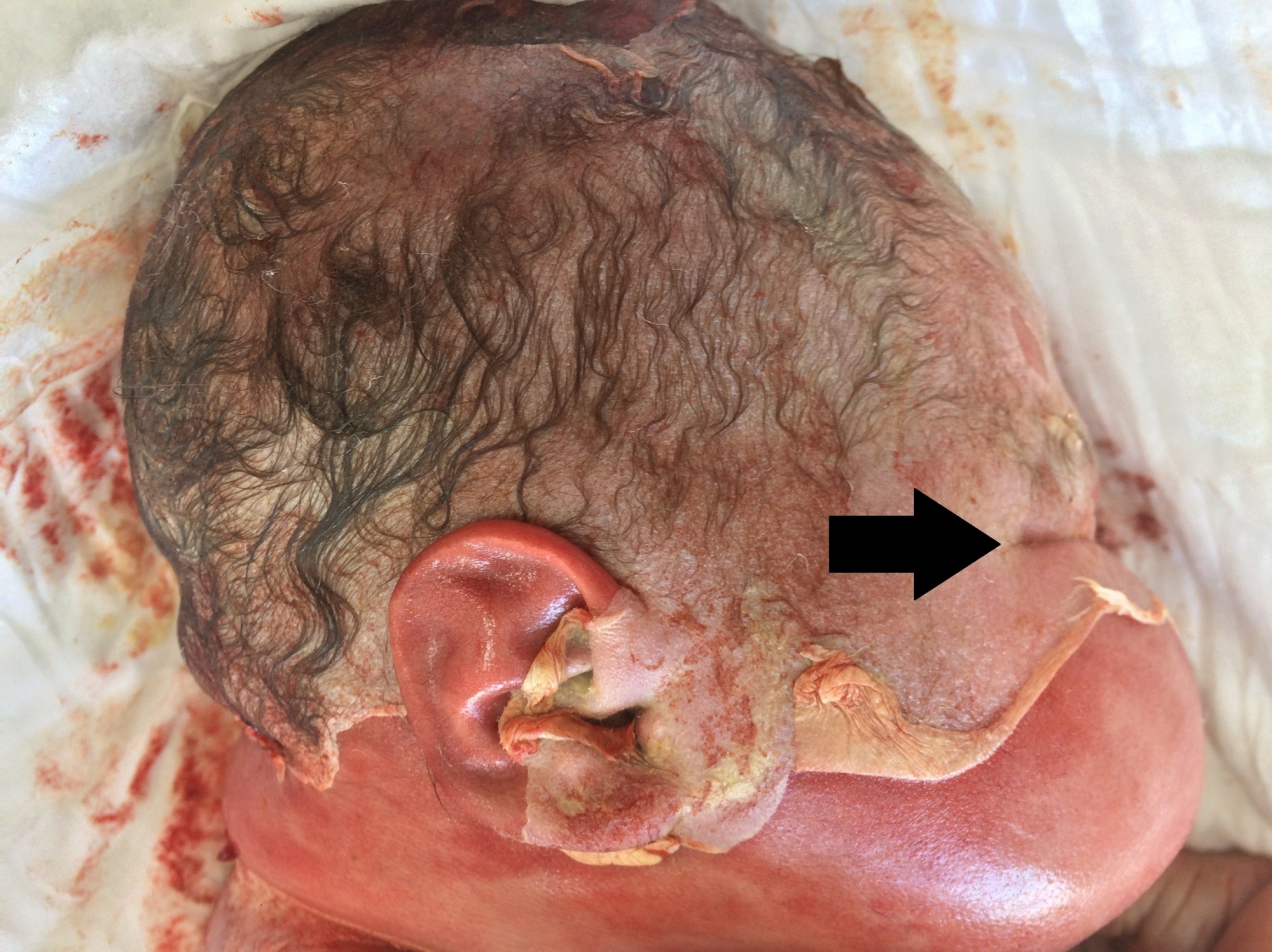
Pathognomonic facial features in fetuses with diabetic fetopathy: slot-like eyelid openings (arrow) and enlarged cheeks. Note: the epidermal peeling due to postmortem intrauterine stay is well-defined.

Upon observation, the fetus was with skin maceration and epidermal detachment from the dermal layer of 70% of the body surface and pathognomonic facial features (Figure 1). The fetus weighed 4.56 kg and had a height of 53 cm with severe generalized adipose tissue aggregation, especially in the face area and a head circumference of 37 cm. Other gross changes, apart from a right-sided clubfoot, were not observed. Upon section generalized organ enlarged for the gestational week was observed, with preserved fetal circulatory openings. Other gross changes were not observed.

**Figure 2:**
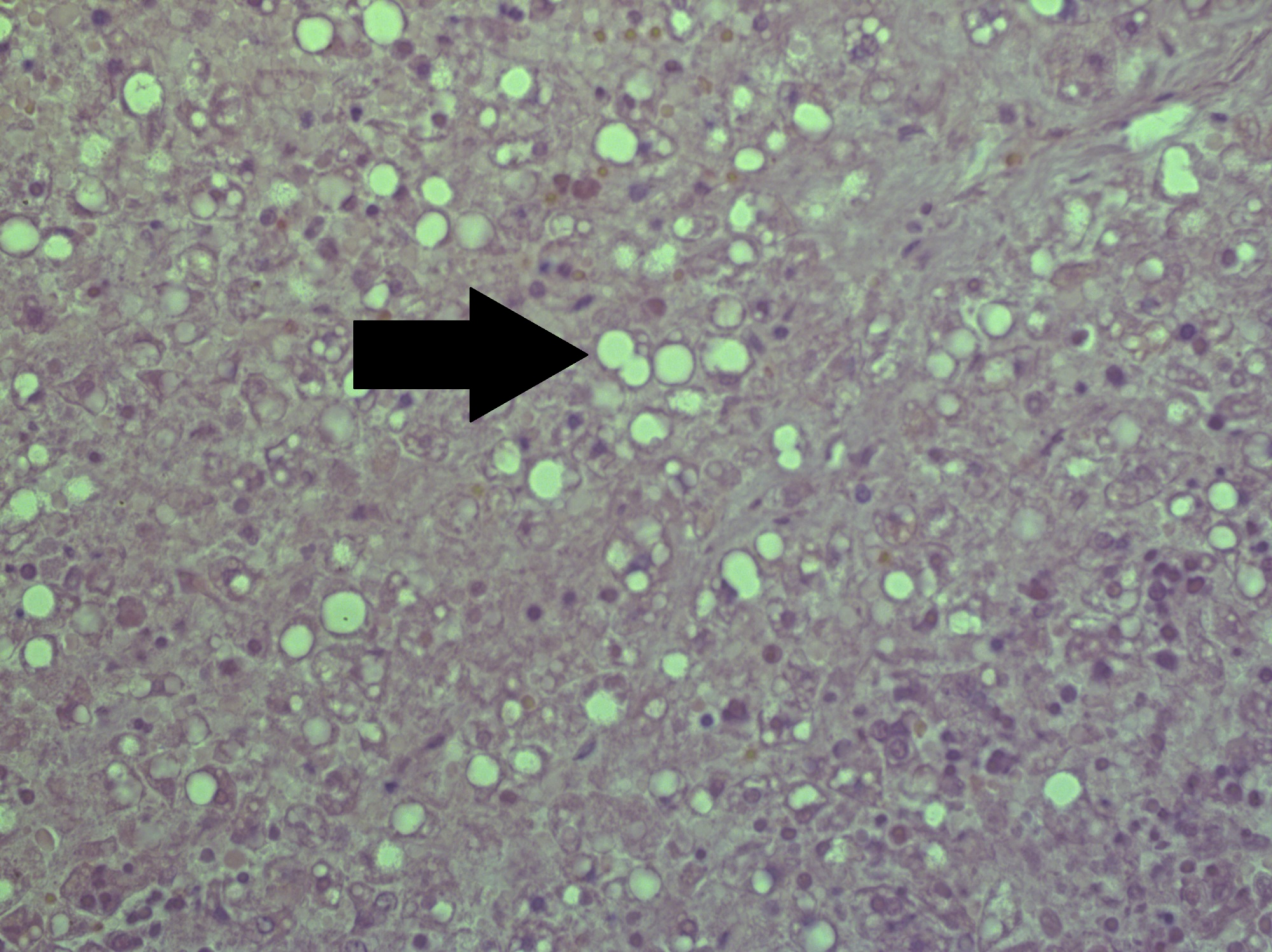
Macrovesicular hepatosteatosis (arrow). Note: severe autolysis is present in parenchyma due to postmortem time to autopsy. Hematoxylin and Eosin stain, original magnification x200.

**Figure 3:**
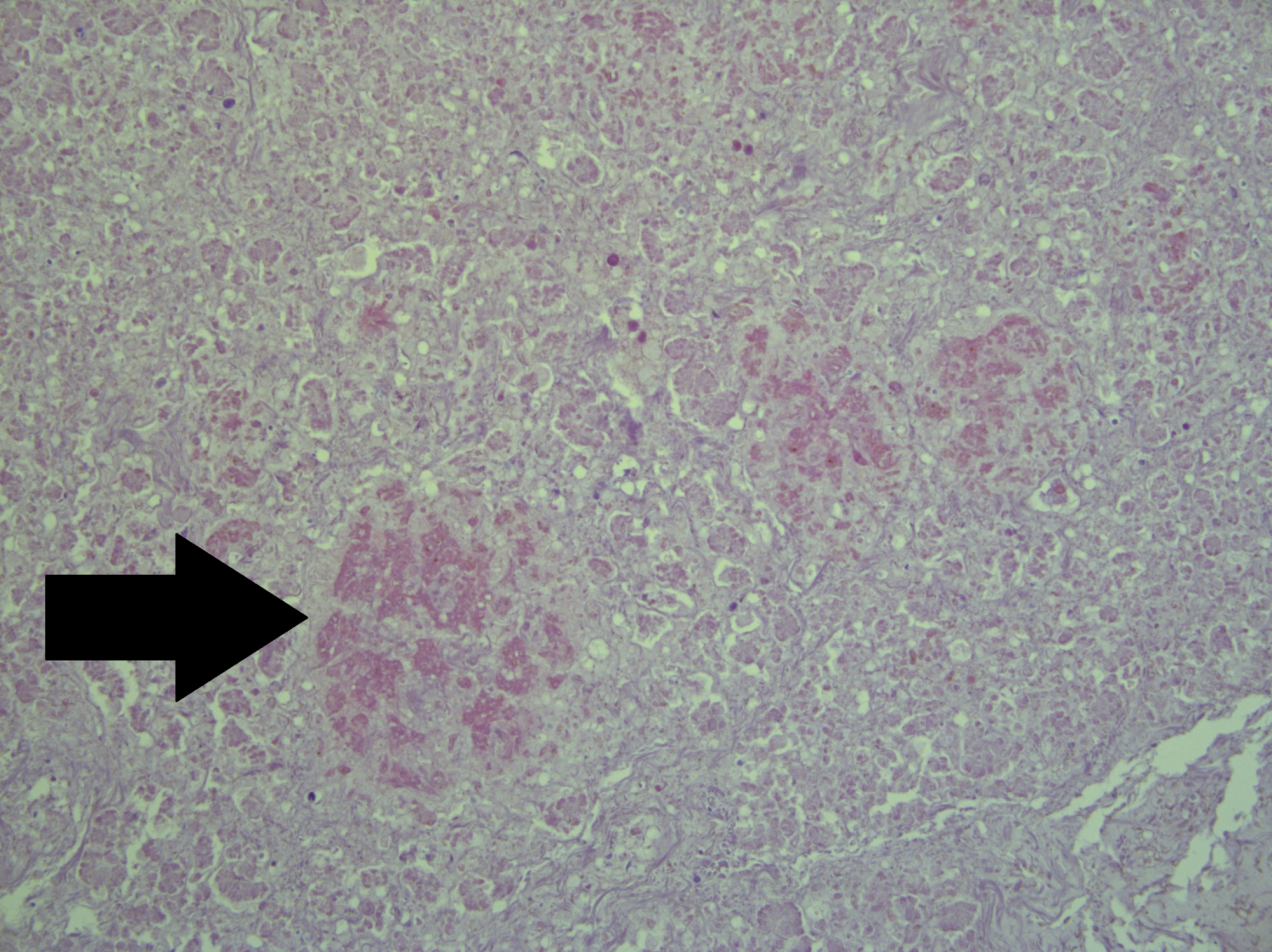
Langerhans isle amyloidosis (arrow) on the background of islet hyperplasia and amylin hypersecretion. Note: severe autolysis is present in parenchyma due to postmortem time to autopsy. Hematoxylin and Eosin stain, original magnification x200.

**Figure 4:**
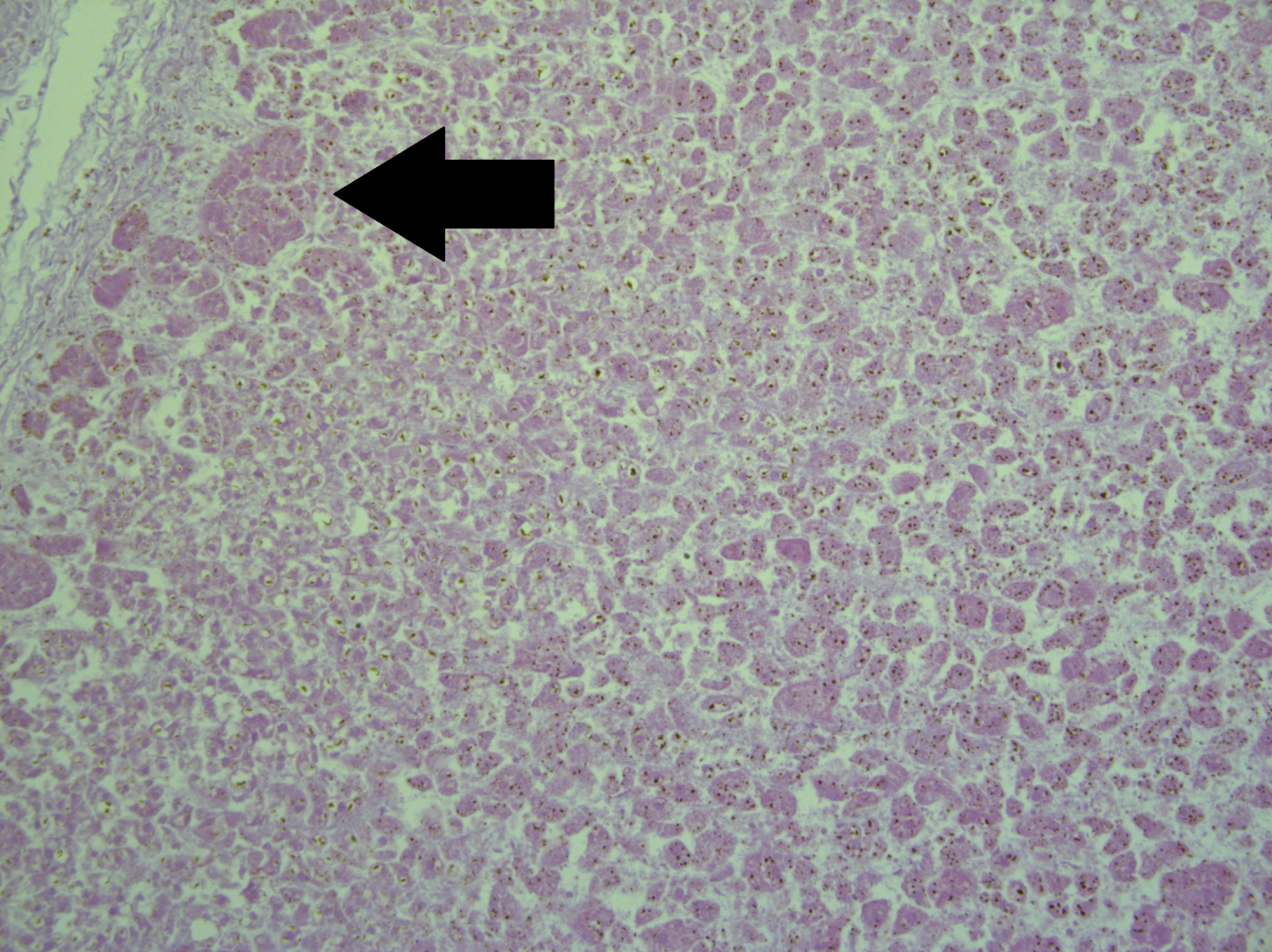
Zona glomerulosa hyperplasia in adrenal glands (arrow). Note: severe autolysis is present in parenchyma due to postmortem time to autopsy. Hematoxylin and Eosin stain, original magnification x200.

On histology, the liver revealed severe generalized macrovesicular steatosis and a number of small cysts (Figure 2). The pancreas revealed not only Langerhans isle hyperplasia, but also Langerhans amyloidosis, evident of the severity of maternal diabetes and fetal hyperglycemia and reactive hyperinsulinemia and increased amylin (islet amyloid precursor protein) secretion, leading to the amyloid depositions (Figure 3). The adrenal glands revealed hyperplasia in zona glomerulosa, due to aldosterone overproduction, evident of fetal hypertension (Figure 4). The kidneys were morphologically intact, apart from a single glomerulus that had fully hyalinised, suggestive of the early development of diabetic microangiopathy. All other histology samples revealed no changes.

Upon request of additional medical history of the mother, no preexisting diabetes or signs of diabetes were established prior to the pregnancy. However, upon admission for the miscarriage her blood sugar levels were 17 mmol/l and within the following week dropped to 10 mmol/l, without anti-diabetic treatment.

Based on the clinical and pathological findings the cause of death was determined as diabetic fetopathy with macrosomia, complicated with fetal malrotation and umbilical cord wrapped around the neck, resulting in intrauterine asphyxia.

## Discussion

Maternal diabetes is one of the most common and dangerous risk factors during pregnancy, as often there are no generalized signs. Gestational diabetes is defined as a new onset of impaired glucose tolerance developing during pregnancy as a result of insulin resistance [1]. Screening programs are helpful in preventing the risk for the fetus and preserving the health of the mother. Seldom, however, even in unmonitored pregnancies can the range of changes affect the fetus in such a way [3].

The current case is an extreme example of an undiagnosed and untreated gestational diabetes mellitus. The changes in the fetal internal organs, such as the Langerhans amyloidosis and the macrovesicular hepatosteatosis, are normal complications of long-lasting diabetes and have so far not been reported in diabetic foetopathy and are evident of the range of fetal changes and fetal impairment of glucose and triglyceride metabolism [6]. In addition, the case is also evident for the duration of fetal hyperglycemia due to maternal diabetes, as in experimental models of type two diabetes and metabolic syndrome, hepatosteatosis starts developing after more than eight weeks of exposure to hyperglycemia, furthermore supporting an alternative thesis of hepatosteatosis as a key factor in the development of insulin resistance and type two diabetes [7,8].

The severity of histological changes in this case is suggestive of further extension of the diagnostic criteria of diabetic fetopathy to include more subtle changes that can be observed clinically and even a combination of maternal-newborn factors, such as blood sugar levels measured every day for the first week and compared between the mother and fetus to establish a dynamic, as well as a similar dynamic with blood pressure, liver enzymes, low-density lipoproteins (LDL), high-density lipoproteins (HDL), potassium, uric acid, creatinine, urea and even pro-inflammatory adipokines.

Such tests with the establishment of maternal-newborn dynamics would be helpful in determining even the slightest postpartum changes and evading further complication to the fetus.

As for histological criteria for the diagnosis of diabetic fetopathy, an argument should be made that the changes sought in miscarried fetuses and deceased newborns should be the same as those sought after in adult patients with long-lasting diabetes.

## Conclusions

The present case illustrates the wide range of severe fetal changes in undiagnosed and untreated maternal diabetes. Although such severe changes rarely develop, their correlation with clinically observed phenomena, suggests the need for expansion of maternal monitoring in cases of gestational diabetes and introduction of new markers and maternal-fetal correlation of such markers postpartum.
